# Psychological factors and obesity, not thyroid biomarkers, predict thyroid-dependent quality of life in treated hypothyroidism: a cross-sectional study

**DOI:** 10.1186/s12902-025-01962-9

**Published:** 2025-05-26

**Authors:** Bence Bakos, Tibor Solymosi, Balázs Szili, Ágnes Vincze, Szilvia Mészáros, Márk Stempler, Richárd Ármós, András Kiss, Anna Bakos, Nikolette Szücs, Péter Reismann, Judit Tőke, Péter Pusztai, Péter Lakatos, István Takács

**Affiliations:** 1 Department of Internal Medicine and Oncology, Semmelweis University, 1083 Korányi Sándor U. 2/a, Budapest, Hungary; 2 Department of Haematology and Internal Medicine, Semmelweis University, 1088 Szentkirályi U. 46, Budapest, Hungary; 3 Department of Clinical Psychology, Semmelweis University, 1091 Üllői Út 25, Budapest, Hungary

**Keywords:** Hypothyroidism, Quality of life, Somatosensory amplification, Depression, Obesity, Reverse-T3

## Abstract

**Background:**

A significant number of patients with primary hypothyroidism report persistent symptoms and decreased quality of life (QoL) despite biochemically adequate levothyroxine replacement. Individual variations in thyroxine conversion, autoimmune inflammation, and psychological factors have all been implicated as a potential cause.

**Methods:**

In this cross-sectional study we have examined the association of numerous demographic, disease-specific, and laboratory parameters as well as three patient reported outcome measures with thyroid-dependent QoL as measured by the Underactive Thyroid-Dependent Quality of Life Questionnaire. Patients were stringently selected to minimize the confounding effect of comorbidities or inadequate hormone replacement. We used validated questionnaires to assess somatosensory amplification, depression, and symptom number. Determinants of QoL were evaluated using uni- and multivariable linear modeling, and mediation analysis.

**Results:**

Our final sample consisted of 157 patients. 70.7% had Hashimoto’s, whereas 29.3% had iatrogenic hypothyroidism. Mean age was 49.5 ± 14.5 years, disease duration: 11.2 ± 8.2 years, thyroxine dose: 1.2 ug/kg bodyweight, TSH: 1.8 ± 0.9 mIU/L. Thyroid-specific biomarkers including TSH, FT3, FT4, rT3, SPINA-GD, anti-TPO, and SHBG had no association with thyroid-dependent QoL. Somatosensory amplification was a strong predictor of the presence and perceived bother of the most common hypothyroidism-associated symptoms. In our final multivariable model (r^2^ = 0.31) the factors associated with thyroid-dependent QoL were somatosensory amplification (*p* = 0.002), BMI (*p* = 0.021), and depression (*p* < 0.001).

**Conclusion:**

These results suggest that psychological factors, particularly somatosensory amplification, might play a major role in influencing QoL in hypothyroid individuals on adequate levothyroxine replacement. Our findings do not corroborate a significant role for autoimmune inflammation or tissue-level hypothyroidism.

## Background

Levothyroxine (LT4) monotherapy with the aim of normalizing serum thyrotropin (TSH) is the mainstay of care for primary hypothyroidism. While symptoms and life expectancy improve markedly with treatment, at least 10–15% of patients report persistent symptoms and reduced quality of life (QoL) despite normalization of TSH [1, 2]. The reasons behind this phenomenon are not completely understood.

One line of thinking emphasizes decreased tissue triiodothyronine (T3) availability with LT4 monotherapy due to individual differences in deiodinase enzyme (D1-3) activity [3]. Results from animal studies [4] suggest that normalization of TSH does not necessarily signal appropriate T3 availability in all tissues. Rodent studies also demonstrated highly variable tissue T3 levels with T4 supplementation alone [5]. Decreased free T3 (FT3) levels have been observed in individuals treated with thyroxine [6], and symptomatic improvement has been reported with normalization of FT3, even in the context of normal TSH levels [7]. Polymorphisms of deiodinase genes have been implied to affect clinical response to T3 replacement [8], and inherited D1 deficiency in humans has also been recently described [9]. Hypothalamic-pituitary thyrotropic activity appears to show individual variability, influenced by several factors, potentially through their effect on hypothalamic D2 activity [10].

Based on these findings, several authors advocate for combined T3 + T4 therapy or T4 monotherapy with lower TSH targets in patients with persistent complaints. While there is some data in favor of these approaches, most randomized studies have failed to show clear benefits [11].

Scientific inquiry into the variability of deiodination was probably the origin of alternative medicine claims for the utility of reverse-T3 (rT3) measurement in managing hypothyroidism. Several websites and books advocate that increased rT3 levels could signify or cause tissue level hypothyroidism. Although these assertions are refuted by the relevant clinical literature, the volume of rT3 testing seems to be increasing in the last decade [12].

Another group of theories focuses on the role of autoimmune inflammation as a potential driver of persistent symptoms independently of thyroid function [13]. Patients with chronic autoimmune thyroiditis generally report lower QoL [14, 15] and more persistent symptoms [16]. Some studies also found an inverse relationship between thyroid peroxidase antibody (aTPO) levels and QoL [13]. In one randomized study thyroidectomy improved QoL in Hashimoto’s patients [17]. Interventions aimed at decreasing aTPO, however, have not been consistently shown to simultaneously improve QoL [18].

Certain authors propose that psychological factors are the main drivers of decreased QoL in hypothyroid patients [19]. They emphasize the aspecific nature of persistent symptoms, such as fatigue and brain fog, and point out that many studies demonstrating a high frequency of these [20] had a selection bias for more dissatisfied patients. Medically unexplained physical symptoms, often attributed to somatic symptom disorder, are common in the general population [21–23]. Given the high prevalence of both conditions, a significant proportion of individuals with somatic symptom disorder are bound to have hypothyroidism causally unrelated to their symptoms. Increasing medicalization of such complaints might also contribute to the growing number of levothyroxine prescriptions that is seen worldwide [24]. Another significant psychological factor might be the medical diagnosis itself, as labeling of hypothyroidism was shown to be associated with worse self-reported health than undiagnosed disease [25].

Given the high prevalence of hypothyroidism and the fact that both under- and overtreatment pose a considerable health risk, the debate on the determinants of QoL in hypothyroidism is eminently important [26]. In this cross-sectional study we aimed to assess known and hypothesized predictors of QoL in a sample of hypothyroid individuals under biochemically optimal LT4 treatment.

## Methods

### Patients and study design

We screened all hypothyroid patients presenting for follow-up at our university outpatient clinic between August 2021 and August 2022. Inclusion and exclusion criteria are shown in Table 1.

**Table 1:** Inclusion and exclusion criteria of the study

Inclusion criteria	Exclusion criteria
Age > 18 years	Signs of acute illness during screening
Primary hypothyroidism of any etiology	Inability to understand and consent to the study
Disease duration > 2 years	Pregnancy
Normal TSH (0.5–5 mIU/L) for > 6 months	Any significant comorbidity likely to affect QoL:
Levothyroxine monotherapy for > 6 months	chronic liver disease
Stable LT4 dose for > 6 months	chronic kidney disease
	organic heart disease
	history of stroke or TIA
	peripheral artery disease
	active malignancy
	systemic autoimmune condition
	diabetes treated with insulin
	morbid obesity (BMI > 40)
	adrenal insufficiency
	other endocrinopathy
	previous diagnosis of any mental illness

As a result of excluding most comorbidities, the only medications used by the participants in addition to LT4 were antihypertensives, gastric acid reducers (PPIs, H2 blockers), oral anti-diabetics, and vitamin D and calcium supplements.

The single visit consisted of a review of the patients’ history, physical examination, blood sampling, and administration of self-reported questionnaires by a dedicated study team. Based on power calculations a sample size of ≥ 138 individuals was planned, giving 95% power to detect an effect of f^2^ ≥ 0.15 with 5 tested predictors and a two-tailed α of 0.05. The study was approved by the National Scientific and Ethical Committee of the Hungarian Medical Research Council (No.: 38233–1/2019/EKU) and was conducted in accordance with the World Medical Association’s Declaration of Helsinki. Written informed consent was obtained from all participants before entering and prior to all study-related procedures. The protocol was pre-registered at clinicaltrials.gov (Id.: NCT05015725, registration date: 2021.06.30.).

### Laboratory testing

TSH-, FT4-, FT3-, and sex hormone binding globulin (SHBG) measurements were undertaken using chemiluminescence immunoassays on the Atellica IM analyzer (Siemens Healthcare Diagnostics Inc., Erlagen, Germany). The linear range of these assays were 0.008–150.0 mIU/L, 1.3–154.8 pmol/L, 0.31–30.80 pmol/L, and 1.6–180.0 nmol/L for TSH, FT4, FT3, and SHBG, respectively.

Anti-TPO was measured using an electrochemiluminescence immunoassay on the Roche Cobas e, platform (Roche Diagnostics, Mannheim, Germany) with a measuring range of 9–600 IU/mL. Reverse-T3 was measured by radioimmunoassay (Diasource Immunoassays, Nivelles, Belgium) with a linear range of 0.02—2.14 ng/mL.

To further assess peripheral thyroid hormone metabolism, the FT3/FT4 and rT3/FT3 ratios were calculated and total peripheral deiodinase activity (GD) was estimated using SPINA Thyr 4.2 (Structure Parameter Inference Approach by Johannes W. Dietrich, Lab XU44, Bergmannsheil University Hospitals, Ruhr University of Bochum, NRW, Germany) [27].

To screen for any intercurrent conditions and to assess proposed markers of tissue level hypothyroidism [28], additional laboratory tests were performed using automated enzymatic methods on a Beckman Coulter AU 5800 analyzer (Beckman Coulter, Brea, USA). These included aspartate aminotransferase, alanine aminotransferase, gamma-glutamyl transferase, bilirubin, sodium, potassium, creatinine, estimated glomerular filtration rate (eGFR), creatine kinase (CK), triglicerides, cholesterol, low-density lipoprotein (LDL), and high-density lipoprotein (HDL).

### Questionnaires

We applied validated, patient-reported questionnaires to assess disease-specific quality of life and other psychometric measures of interest. The Underactive Thyroid-Dependent Quality of Life Questionnaire (ThyDQoL) and Underactive Thyroid Symptom Rating Questionnaire (ThySRQ) [29, 30] were used to measure the perceived effect of hypothyroidism on QoL and the number of hypothyroid symptoms. These instruments were translated into Hungarian during a three phase linguistic validation process described in detail elsewhere [31].

ThyDQoL assesses disease-specific QoL in eighteen domains. Average Weighted Impact Scores between −9 and 3 can be calculated taking into account all 18 (AWI–18), or 14 (AWI-14) domains with a relative weight of importance allocated by the patient. Energy, Weight, Bodily discomfort, and Depression, that are also part of ThySRQ, are excluded from AWI-14. With the simultaneous use of ThySRQ, we have used this later score as our main outcome measure.

ThySRQ is a fifteen-item inventory on the presence and perceived bother of hypothyroidism-related symptoms. The number of symptoms experienced by each participant and mean bother rating for each individual symptom (ranging between 0—3) was recorded. As the questionnaire showed no clear factor structure during earlier studies [32], no weighted total score was calculated.

Depressive symptoms and negative affective state were evaluated using the nine-item Patient Health Questionnaire-9 (PHQ-9) [33], which has a total outcome score between 0–27. We used the ten-item Somatosensory Amplification Scale (SSAS) [34], which has a potential mean outcome score between 1–5, to quantify individual somatosensory amplification. These latter two instruments already had a Hungarian version available at the time of this study [35, 36].

### Statistical methods

Continuous variables are reported as means ± standard deviations, categorical variables as percentages. Formal testing of distributions and homoscedasticity were performed using Kolmogorov–Smirnov (K-S) test and Levene’s test, respectively. Where violation of assumptions was suspected, we used robust statistical methods including bootstrapping (bias-corrected and accelerated (BCa)) and heteroscedasticity-consistent correction of standard errors to minimize bias.

Between-group differences were explored using ANOVA for continuous-, and chi-squared test or Fisher's exact test for categorical outcomes. Relationships between continuous variables were examined using bivariate Pearson correlation and linear regression analysis. Missing data was handled by listwise deletion.

Candidate predictors of thyroid-dependent QoL were screened with univariable tests. Variables showing significant univariable association were entered simultaneously in a multivariable backwards elimination model with removal criteria of *p* > 0.05. For predictors that retained significance in this second model, potential interactions and mediation pathways were further explored.

Questionnaire reliability was estimated using Cronbach's alpha. Construct validity was verified with confirmatory factor analysis (CFA) for instruments with a previous Hungarian version. Exploratory factor analysis (EFA) was repeated for ThyDQoL after linguistic validation. Factor analysis was omitted for ThySRQ as no clear factor structure was previously demonstrated for this instrument.

The threshold of statistical significance was set at *p* < 0.05 in all analyses. Bonferroni correction was used to correct for familywise error rate inflation where applicable. Analyses were performed using IBM SPSS Statistics for Windows (version 28.0, Released 2021, IBM Corp, Armonk, NY), Process Macro version 4.0 [37], and JASP (version 16.4.0, JASP Team, University of Amsterdam).

## Results

### Patient characteristics

We screened 262 consecutive patients treated with LT4 monotherapy at our outpatient center. Based on the above detailed criteria, 105 individuals were excluded: 8 due to non-standard (e.g. secondary) etiology of hypothyroidism, 24 due to abnormal TSH and/or change in LT4 dose in the 6 months prior to the study, 38 due to relevant comorbidities, 30 due to abnormal TSH on screening, and 5 due to incomplete questionnaire data. This left us with a sample of 157 participants. Patient characteristics compared by etiology of hypothyroidism are shown in Table 2.

**Table 2:** Patient characteristics at screening by etiology of hypothyroidism

	Autoimmune thyroiditis	Iatrogenic hypothyroidism	*p* value
No. of participants	111 (70.7%)	46 (29.3%)	
Sex			
Female	104 (93.7%)	38 (82.6%)	**0.04**
Male	7 (6.3%)	8 (17.4%)	
Age (years)	47.5 ± 15.0	54.1 ± 12.0	**0.009**
Duration of hypothyroidism (years)	10.2 ± 6.7	13.7 ± 10.7	0.052
Median no. of co-existing medical conditions	1	1	
Median no. of co-medications	1	1	
BMI (kg/m^2^)	25.4 ± 4.6	26.5 ± 4.4	0.116
LT4 dose/bodyweight (ug/kg)	1.1 ± 0.4	1.2 ± 0.4	0.152
TSH at screening (mIU/L)	1.8 ± 0.9	1.7 ± 0.8	0.350
No. of hypothyroidism-related symptoms (ThySRQ)	6.0 ± 3.2	5.5 ± 3.0	0.396
Depression (PHQ-9)	7.1 ± 5.3	5.7 ± 4.2	0.075
Somatosensory amplification (SSAS)	2.7 ± 0.7	2.6 ± 0.5	0.423

### Parameters of thyroxine metabolism

TSH was dependent on FT4 (F(1,154) = 13.2, β = −0.28, *p* < 001), but not on FT3 (F(1,154) = 1.9, β = −0.11, *p* = 0.12) or rT3 (F(1,154) = 2.0, β = −0.11, *p* = 0.13). Higher rT3 was associated with a lower FT3/FT4 ratio (F(1,154) = 13.2, β = −0.28, *p* = 0.002). After correction with TSH, levothyroxine dose (ug/kg) predicted FT4 (F(2,153) = 14.7, r^2^ = 0.16, β = 0.29, *p* < 0.001), but not FT3 (F(2,153) = 1.3, r^2^ = 0.02, β = 0.07, *p* = 0.38) or rT3 (F(2,153) = 1.7, r^2^ = 0.02, β = 0.1, *p* = 0.24).

Potential markers of tissue level hypothyroidism - SHBG (F(1,154) = 0.001, β = −0.003, *p* = 0.97), CK (F(1,154) = 1.23, β = 0.09, *p* = 0.27), eGFR (F(1,154) = 1.4, β = 0.09, *p* = 0.23), total cholesterol (F(1,154) = 0.18, β = −0.4, *p* = 0.66), and LDL (F(1,154) = 0.83, β = −0.7, *p* = 0.34) - showed no association with the FT3/FT4 ratio.

### Patient reported outcomes

SSAS, PHQ-9, and ThySRQ showed adequate internal consistency (Cronbach's alpha: 0.735–0.833). CFA verified a clear one-factor structure for both SSAS (CFI = 0.977, RMSEA = 0.049) and PHQ-9 (CFI = 0.998, RMSEA = 0.034). The frequency and mean bother for each symptom is shown in Table 3. There was a very strong correlation between bother and frequency (r = 0.985, *p* < 0.001). Only 4.5% of patients reported no symptoms at all.

**Table 3:** Frequency and bother rating of ThySRQ symptoms, and their association with somatosensory amplification. (Due to Bonferroni correction for multiple testing *p* < 0.0033 is considered to be significant.)

	Frequency	Bother rating	Correlation with SSAS	
	%	M ± SD	r	p
Tiredness	81	1.7 ± 1.1	0.26	**< 0.001**
Feeling depressed/low	71	1.5 ± 1.2	0.24	**0.002**
Skin problems	56	1.2 ± 1.3	0.33	**< 0.001**
Hair problems	51	1.2 ± 1.4	0.08	0.31
Memory problems	47	1.1 ± 1.3	0.23	0.004
Feeling cold	42	0.8 ± 1.1	0.24	**0.003**
Difficulty concentrating	41	1.0 ± 1.3	0.28	**< 0.001**
Dizziness/lightheadedness	40	0.9 ± 1.2	0.27	**< 0.001**
Weight gain	30	0.7 ± 1.2	0.09	0.25
Nail problems	27	0.6 ± 1.1	0.22	0.005
Voice problems	24	0.5 ± 0.9	0.18	0.02
Constipation	22	0.4 ± 0.9	0.06	0.46
Speech problems	22	0.5 ± 1.1	0.22	0.007
Hearing problems	17	0.3 ± 0.9	0.16	0.05
Loss of appetite	13	0.1 ± 0.5	0.10	0.20

### Quality of life

**Fig. 1:**
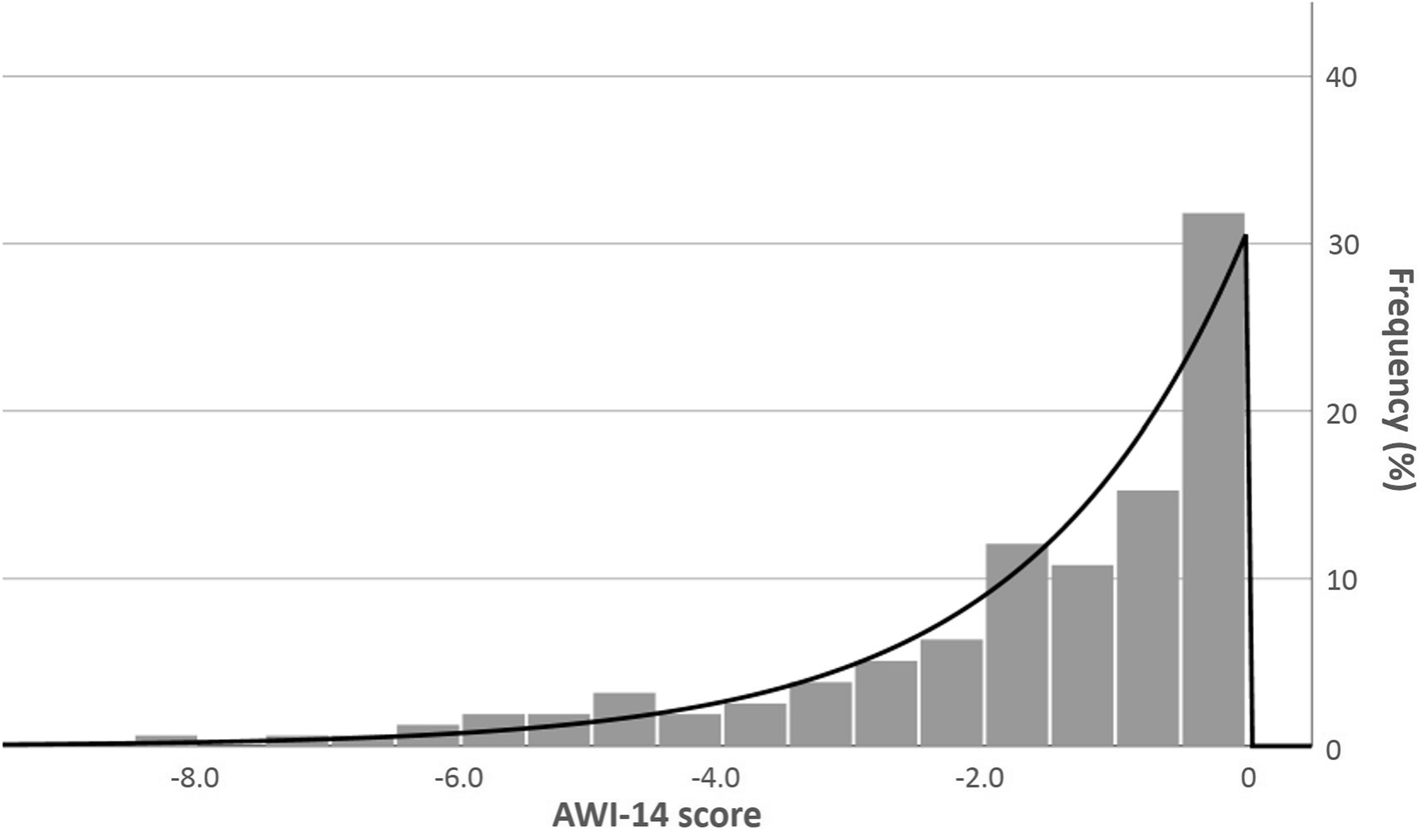
Distribution of AWI-14 scores in our sample, with exponential curve shown. Visual inspection of the histogram, but not the K-S test (*p* < 0.001), suggested an exponential distribution

Both the 18- and the 14-item ThyDQoL had a high internal consistency (Cronbach's alpha = 0.961 and 0.940 respectively). Principal component extraction with varimax rotation yielded a single-factor solution based on the scree plot. This explained 54.4% of the variance, with all items showing satisfactory loading (> 0.6). AWI–14 scores in our sample ranged between −8.4 and 0 with a mean of −1.63 (± 1.8) and a mode of 0. The distribution differed from normal with a significant negative skew. Only 14.6% and 6.4% of scores were below −3.4 (mean-1SD) and −5.2 (mean-2SD) respectively. Visual inspection of the histogram suggested an exponential distribution (Fig. 1), however, this was not supported by the K-S test (*p* < 0.001) or P-P plot.

Potential determinants of AWI-14 including medical history as well as demographic-, psychometric-, and laboratory factors were first assessed in univariable analysis (Table 4).

**Table 4:** Results from the univariable analysis with AWI-14 score as the dependent variable

	Predictor	B	95% CI of B (BCa)	F	*p* value	η^2^
Demographic data	Sex (female vs. male)	−0.90	−1.57—(−0.08)	3.63	**0.023**	0.023
	Age (years)	0.010	−0.007—0.03	0.98	0.24	0.006
	Level of education	N/A	N/A	0.77	0.51	0.015
	Marital and family status	N/A	N/A	0.34	0.85	0.009
	Geographic location	N/A	N/A	0.37	0.83	0.01
	Employment status	N/A	N/A	1.20	0.31	0.04
	Financial situation	N/A	N/A	1.48	0.21	0.04
Medical history	Etiology (autoimmune vs. iatrogenic)	−0.56	−1.06—(−0.06)	3.38	**0.029**	0.021
	Disease duration (years)	0.002	−0.03—0.03	0.02	0.85	0.00
	No. of comorbidities	−0.22	−0.49—0.37	2.50	0.11	0.016
	No. of co-medications	−0.13	−0.36—0.05	1.49	0.19	0.01
	LT4 dose (ug/kg bodyweight)	−0.30	−1.07—0.61	0.68	0.51	0.004
	BMI	−0.10	−0.17—(−0.38)	11.81	**< 0.001**	0.071
Questionnaire data	Somatosensory amplification (SSAS)	−1.12	−1.56—(−0.68)	28.93	**< 0.001**	0.16
	Depression (PHQ-9)	−0.17	−0.23—(−0.11)	45.68	**< 0.001**	0.23
	No. of symptoms (ThySRQ)	−0.26	−0.34—(−0.17)	40.0	**< 0.001**	0.205
Laboratory parameters	TSH (mIU/L)	−0.14	−0.50—0.25	0.44	0.45	0.005
	FT4	0.10	−0.025—0.25	1.91	0.14	0.012
	FT3	0.114	−0.11—0.5	0.6	0.25	0.004
	FT3/FT4 ratio	−0.38	−6.10—4.25	0.02	0.85	0.0
	SPINA-GD	−0.004	−0.064—0.038	0.19	0.86	0.0
	rT3	0.53	−0.86—2.02	0.38	0.47	0.002
	rT3/FT3 ratio	1.40	−4.49—7.22	0.15	0.6	0.001
	SHBG	0.002	−0.005—0.008	0.30	0.57	0.002

Patients with chronic autoimmune thyroiditis reported a greater negative impact of hypothyroidism on QoL, as did females, and participants with higher BMI. Somatosensory amplification (SSAS), depression (PHQ-9) and the number of hypothyroidism-related symptoms (ThySRQ) were also significant predictors. None of the thyroid-related biomarkers were associated with AWI-14.

In the subgroup of Hashimoto’s patients AWI-14 had a significant association with the initial TSH levels at the time of diagnosis (F(1,88) = 1.9, β = 0.145, *p* = 0.024) but not with current anti-TPO values (F(1,109) = 0.001, β = 0.003, *p* = 0.97).

Among participants with iatrogenic hypothyroidism mean AWI-14 scores were −1.35 ± 1.34, −1.22 ± 1.54, and −1.096 ± 1.00 for patients with a history of thyroid cancer, thyroidectomy, and radioiodine treatment, respectively (F(2,43) = 0.088, η^2^ = 0.004, *p* = 0.92).

The six significant univariable predictors of AWI-14 were entered into a multivariable backwards elimination model with removal criteria of *p* > 0.05. Sex (*p* = 0.32), etiology (*p* = 0.09), and symptom number (ThySRQ) (*p* = 0.07) were removed in three consecutive steps, which left depression (PHQ-9), somatosensory amplification (SSAS), and BMI as significant predictors of thyroid-dependent QoL in the final model (Table 5). Moderation analysis revealed no significant interactions.

**Table 5:** Final multivariable model of significant predictors of AWI-14 after stepwise backwards elimination. (F(3,148) = 21.79, *p* < 0.001, r^2^ = 0.31 for the whole model)

Predictor	B	95% CI of B (BCa)	*p* value	η^2^
BMI (kg/m^2^)	−0.07	−0.13—(−0.01)	0.021	0.046
Somatosensory amplification (SSAS)	−0.68	−1.11—(−0.27)	0.002	0.064
Depression (PHQ-9)	−0.12	−0.19—(−0.05)	< 0.001	0.113

**Fig. 2:**
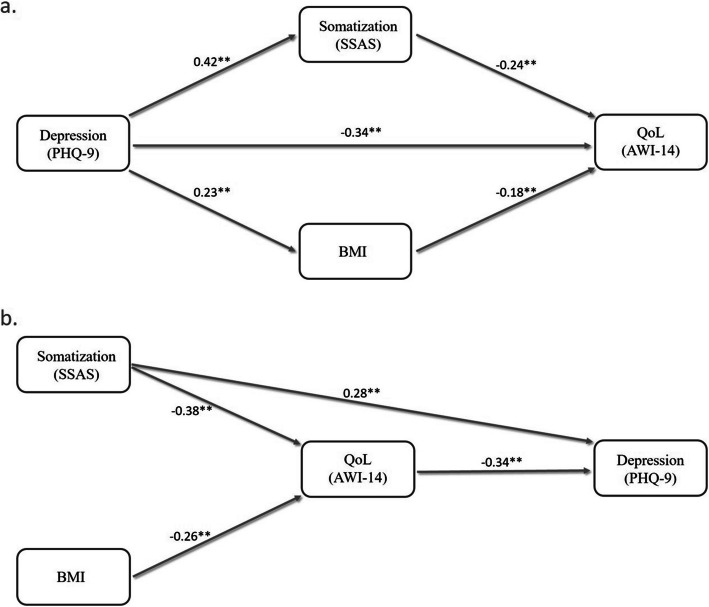
Mediation models exploring potential pathways between variables that showed a significant multivariable association with AWI-14. Model a. considers both depression (PHQ-9) and somatosensory amplification (SSAS) to be antecedent to AWI-14. Model b. considers somatosensory amplification to be antecedent and depression to be consequent to AWI-14. Standardized effect sizes are presented, non-significant pathways are omitted for clarity. ** *p* < 0.01

Given the interrelatedness of these factors, shared variance and potential indirect pathways were explored in two mediation models (Fig. 2). In the first one (Fig. 2a) both depression and somatosensory amplification were assumed to be antecedent to thyroid-dependent QoL. In the second model (Fig. 2b) depression was conceptualized as consequent to QoL.

The effect of somatosensory amplification on the frequency and bother of individual symptoms have also been explored separately (Table 3).

## Discussion

In this study we have assessed the perceived effect of hypothyroidism on QoL and examined its relationship with a number of biological and psychological factors in a sample of hypothyroid patients under biochemically optimal management, as recommended by current guidelines. The potential confounding effect of comorbidities, co-medications and recent changes in LT4 dose were mitigated by stringent inclusion criteria.

The perceived negative effect of hypothyroidism on QoL was moderate and comparable to earlier studies using the same instruments [29, 32]. AWI-14 scores were heavily skewed towards 0, with a minority of patients reporting larger effects.

### Tissue T3 availability

Reduced QoL despite adequate LT4 replacement is often attributed to variations in peripheral deiodination and tissue T3 availability. In accordance with earlier studies [38], we have found that thyroxine dose was positively associated with FT4-, but not with FT3 levels, with larger LT4 doses resulting in lower FT3/FT4 ratios. TSH was also inversely associated with FT4, but was not dependent on FT3 levels. These results support the notion that LT4 monotherapy primarily normalizes TSH through T4 availability, while serum and potentially tissue FT3 levels may be contingent on other factors [39]. However, TSH (within the normal range), LT4 dose, FT3, FT4, and rT3 levels, as well as the FT3/FT4 and rT3/FT3 ratios and the estimated total peripheral deiodinase activity (SPINA-GD) were not associated with differences in AWI-14 scores in our study. Potential markers of tissue-level hypothyroidism including cholesterol, LDL, SHBG, or CK showed no association with either hypothyroidism-related QoL or the biomarkers of thyroid function.

While these findings do not eliminate the possibility of tissue T3 deficiency as a contributor to decreased QoL in treated hypothyroidism, neither do they support this theory. Our data also indirectly argue against the utility of lower TSH or higher FT3 treatment targets, as these values had no predictive power with respect to symptoms or thyroid-dependent QoL.

Unfounded claims regarding the utility of rT3 in guiding hypothyroidism treatment have been gaining popularity in recent years. Despite refutation of this idea by the relevant literature [12], the number of rT3 orders seems to be increasing. In our study we have demonstrated a lack of association between rT3 levels and the perceived effect of hypothyroidism on QoL in treated hypothyroidism. Being, to the authors’ knowledge, the first formal investigation into the matter, this finding might provide additional help in clarifying the ineffectuality of rT3 testing in this context.

### Autoimmune inflammation

Untreated hypothyroidism is a well-known inflammatory trigger [40]. However, several researchers suggest that low-grade systemic inflammation associated with autoimmunity may contribute to decreased quality of life in patients with Hashimoto’s, irrespective of thyroid function [15, 41]. Endothelial dysfunction [42], microvascular changes [43], and inflammatory involvement of both the central [44] and peripheral [45] nervous systems have been implicated in this process.

While mean AWI-14 scores were lower in the Hashimoto’s group of our study, this effect accounted for only 2% of variance in thyroid-dependent QoL among the patients. Furthermore, etiology did not retain its significant effect after correction for other significant univariable predictors in the multivariable model. We also found no difference in the number of symptoms between patients with Hashimoto’s and patients with iatrogenic hypothyroidism. Additionally, and contrary to some previous works [13], higher aTPO levels were not associated with lower thyroid-dependent QoL among our patients with Hashimoto’s. Taken together these findings argue against the primacy of autoimmune inflammation as the driver of decreased QoL in treated hypothyroidism.

Interestingly, Hashimoto’s patients with higher AWI-14 scores in our sample had milder hypothyroidism (lower TSH) at the time of the initial diagnosis. A number of explanations are possible for this phenomenon. Individual differences in autoimmune inflammation could lead to a more severe clinical phenotype and an earlier diagnosis in certain cases. However, it is equally, if not more likely, that patients with significant psychosocial comorbidities are more frequently assessed for hypothyroidism and are therefore diagnosed earlier in the course of the disease [46].

### Psychosocial comorbidities

#### Depression

Association between patient reported depression and thyroid-specific quality of life is well established [32], and was verified in our study. While the depression domain of ThyDQoL is not used in calculating AWI-14, PHQ-9 was still the strongest individual predictor of low AWI-14 scores. Furthermore, although we excluded all participants with a history of mental illness, 15% of the patients had PHQ-9 values above 10, which can be considered a reasonable cut-off for clinical depression [47]. Several potential mechanisms might contribute to this association. The link between hypothyroidism and depression has been repeatedly demonstrated for centuries [48, 49]. In untreated disease, depressive symptoms are mostly ascribed to the direct neuropsychiatric consequences of thyroid hormone deficiency. However, controversies still remain in patients under medical treatment with LT4 [50].

Besides directly affecting the central nervous system, hypothyroidism may also potentially influence affective state as a consequence of persistent bodily symptoms and decreased QoL (Fig. 2b). A reverse direction of causality (Fig. 2a) is also possible, as individuals with symptoms of unrecognized depression (e.g. changes in weight, appetite, sleep, energy, and mood) might be more frequently evaluated for and diagnosed with hypothyroidism [46]. Consequently, in certain cases, depression and the associated decrease in QoL might be independent from the co-occurring thyroid condition. In such instances hormone replacement in and of itself may be inadequate to address all aspects of health. Based on this assumption, longitudinal studies exploring the usefulness of screening and adjunctive treatment of depression to improve QoL in hypothyroid patients under optimal hormone replacement could be well warranted. Depression and negative affective state are well recognized contributors of decreased quality of life, and treatment of depressive disorders has been shown to carry some benefits in terms of QoL [51]. However, in contrast to studies assessing alternative methods of hormone replacement, such psychologically oriented interventional trials are notably lacking.

#### Somatosensory amplification

Somatosensory amplification describes the individuals’ tendency to notice bodily sensations as unusually intense and label them as harmful [52]. It is a temporally stable psychometric construct with trait-like properties that has been suggested to be a major contributor to the development of medically unexplained persistent somatic symptoms, and somatic symptom disorder [53, 54]. Individuals with higher somatosensory amplification have been shown, in a number of contexts, to experience more physical symptoms and consider these to be more severe and more alarming. Symptom focusing and symptom amplification in turn both contribute to decreased physical and mental capacity and impaired quality of life. Neural correlates underlying these processes have been increasingly elucidated in recent years [55, 56]. The effect of somatization on health-related quality of life has been studied in several contexts [57–59], its potential importance in determining thyroid-dependent QoL, however, has only been suggested very recently [60].

Among our participants SSAS was a strong individual predictor of AWI-14, and its effect remained significant after correction for all other covariates. Somatosensory amplification also predicted symptom number and the presence of several of the most common symptoms (Table 3). The remarkably strong association between the frequency and mean bother of individual symptoms is also highly suggestive of an underlying process affecting both the presence and the interpretation of symptoms.

While the cross-sectional design of our study does not permit to assert causality, there is a strong theoretical basis to consider somatosensory amplification to be antecedent to both symptom number as well as thyroid-specific QoL [34, 60–62].

The potential role of somatosensory amplification in mediating the effect of depression was also assessed (Fig. 2a). In this model most (70%) of the effect of PHQ-9 on AWI-14 was direct, however, the PHQ-9—> SSAS—> AWI-14 mediation pathway was also statistically significant and responsible for 20% of the total effect. This finding is in accordance with the well-established bidirectional relationship between somatization and depression [63, 64].

#### Obesity

Obesity has been associated with decreased QoL in individuals with [65] and without [66] hypothyroidism. The inverse relationship between obesity and health-related quality of life is extensively studied but still incompletely understood. Chronic medical conditions associated with higher BMI, as well as mental and economic consequences of obesity have been implicated as intermediaries.

In our study BMI was a significant predictor of AWI-14 independent of etiology, symptoms, somatosensory amplification, and depression. In the multivariable model where depression was hypothesized to be antecedent to both obesity and thyroid-dependent QoL (Fig. 2b), a small but statistically significant effect was observed for the PHQ-9—> BMI—> AWI-14 mediation pathway, accounting for 10% of the total effect of depression on AWI-14.

Weight loss in overweight patients has been shown to improve health-related QoL in a number of different settings [67]. A few studies have demonstrated a lack of relationship between incidental weight loss and QoL outcomes after the initiation or modification of thyroid hormone replacement [68]. However, investigations into the effect of behavioral or pharmacologic weight loss interventions in patients with treated hypothyroidism are lacking.

## Conclusions

The roles of somatosensory amplification, depression, and obesity in the link between hypothyroidism and decreased QoL are not fully explored, and determining their potential causal influence remains challenging. Nevertheless, when considered collectively, our findings are consistent with the theory that once TSH is normalized, thyroid-dependent QoL is primarily influenced by unrecognized comorbidities such as obesity, somatic symptom disorder, and depression. In cases that would otherwise be further investigated or labeled as medically unexplained symptoms, [22, 69] the concomitant diagnosis of hypothyroidism could readily shift both the patients’ and healthcare providers’ attention towards thyroid dysfunction as the sole cause of all complaints. In such instances, hypothyroidism could be an ideal confounder, given both its high prevalence and the large number of non-specific symptoms associated with the condition.

Interventional trials aimed at improving QoL in hypothyroidism through targeting depression, somatosensory amplification or obesity are lacking. Based on our and others’ results, however, such investigations would be very well warranted. Further expanding research into the psychological domain and assessing factors such as personality, intolerance of uncertainty, health-related beliefs, or locus of control might also prove beneficial in exploring individual differences in QoL among hypothyroid patients.

Pharmacological studies, such as those assessing the effect of combined T3 + T4 replacement, could also benefit from correcting for psychological covariates, especially somatosensory amplification, as these could be significant confounders.

Our study had a number of strengths. The first is the relatively large sample size and the comparison of various etiologies. The second is the stringent exclusion criteria used to minimize the confounding effect of known comorbidities and suboptimal LT4 replacement. The third is the use of valid and reliable patient reported outcomes in conjunction with standard laboratory testing. Of note, to the authors’ knowledge this is the first study to assess the role of somatosensory amplification and reverse-T3 testing in this context.

The main weakness of our work is its cross-sectional design which greatly limits inferences of causality and necessitates further prospective validation of our results.

## Data Availability

The datasets used and/or analysed during the current study are available from the corresponding author on reasonable request.

## Abbreviations

anti-TPO/aTPO: Thyroid peroxidase antibody
AWI-14: Average weighted impact score based on 14 domains
AWI-18: Average weighted impact score based on 18 domains
BCa: Bias-corrected and accelerated
BMI: Body mass index
CFA: Confirmatory factor analysis
CK: Creatine kinase
D1-3: Type I-III deiodinase enzyme
EFA: Exploratory factor analysis
eGFR: Estimated glomerular filtration rate
FT3: Free triiodothyronine
FT4: Free thyroxine
GD: Estimated total peripheral deiodinase activity
H2: Histamine H2-receptor
LDL: Low-density lipoprotein
LT4: Levothyroxine
PHQ-9: Patient health questionnaire-9
PPI: Proton pump inhibitor
QoL: Quality of life
rT3: Reverse triiodothyronine
SHBG: Sex hormone binding globulin
SSAS: Somatosensory amplification scale
SPINA: Structure parameter inference approach
T3: Triiodothyronine
T4: Thyroxine
ThyDQoL: Underactive Thyroid-Dependent Quality of Life Questionnaire
ThySRQ: Underactive Thyroid Symptom Rating Questionnaire
TSH: Thyrotropin
